# Fibrous dysplasia, shepherd’s crook deformity and an intra-capsular femoral neck fracture

**DOI:** 10.1007/s11751-013-0174-7

**Published:** 2013-09-14

**Authors:** Louay Al-Mouazzen, Karthig Rajakulendran, Nurul Ahad

**Affiliations:** 1 ST3 Trauma & Orthopaedics Cheltenham General Hospital, Gloucestershire, UK; 2 ST4 Trauma & Orthopaedics Royal National Orthopaedic Hospital, Stanmore, UK; 3 Queen’s Hospital, Rom Valley Way, Romford, Essex, UK

**Keywords:** Fibrous dysplasia, Shepherd’s crook, Femoral neck fracture

## Abstract

Fibrous dysplasia (FD) is a rare bone disorder in which normal medullary bone is replaced by fibro-osseous tissue. It typically presents in childhood with pain, skeletal deformities, gait abnormalities and occasionally, fatigue fractures. The management of FD remains a challenge. Surgical procedures have been developed to provide symptom relief, correct skeletal deformity and offer mechanical support in cases at risk of fracture. However, there is a paucity of data on the management of acute femoral neck fractures in the adult population with FD. We report the case of a 23-year-old man with a shepherd’s crook deformity secondary to FD, who sustained an intra-capsular femoral neck fracture whilst playing football. The patient initially underwent closed reduction and internal fixation with cannulated screws. However, during the procedure, a guide wire broke whilst inside the femoral head. The patient was referred to the senior author, who undertook a second operation to remove the metalwork and correct the varus deformity using a closing-wedge femoral osteotomy, whilst achieving osteosynthesis at the fracture site. At 1-year follow-up, the patient is pain-free and demonstrates a full range of movement. These cases can be technically demanding and carry a greater risk of complication. It is important that preoperative planning is undertaken and surgery performed by individuals with experience in managing FD and complex femoral neck fractures. Correction of the skeletal deformity whilst fixing the fracture will help restore the mechanical axis and reduce the risk of a recurrent fracture.

## Introduction

Fibrous dysplasia (FD) is a benign bone disorder characterised by the pathological replacement of normal bone with fibro-osseous tissue. The natural history of FD is variable, but most cases present in late childhood and adolescence. It can manifest in a monostotic or polyostotic form, causing pain, skeletal abnormalities, such as the shepherd’s crook deformity and chronic fatigue fractures. Various surgical procedures to manage FD lesions have been described, with mixed results. However, there is little in the literature detailing the management of acute femoral neck fractures in the adult population with FD.

We report the case of a 23-year-old man with a shepherd’s crook deformity secondary to fibrous dysplasia, who sustained an intra-capsular femoral neck fracture.

## Case report

**Fig. 1:**
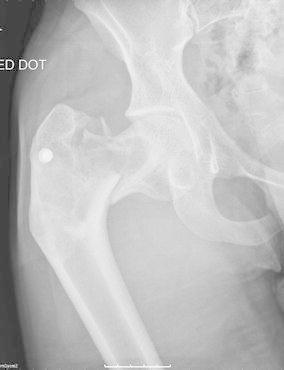
Antero-posterior radiograph of the right hip showing a shepherd’s crook deformity with an intra-capsular femoral neck fracture

A 23-year-old man presented to the emergency department with severe right hip pain, following a fall whilst playing football. Clinical examination demonstrated shortening of the right limb with painful hip movements and an inability to weight bear. Plain radiographs of the hip revealed an intra-capsular femoral neck fracture on a background of fibrous dysplasia with a shepherd’s crook deformity (Fig. 1). The radiographs also showed evidence of a previous proximal femur fracture. On further questioning, the patient informed us that 2 years ago, he had sustained a right femoral fracture following a fall in Pakistan. He was treated non-operatively, with skeletal traction and bed rest for 6 weeks. The hip pain had gradually improved, and he was able to resume some sporting activity, but at a lower functional level. Furthermore, he reported a persistent limp and a leg length discrepancy since the original injury.

**Fig. 2:**
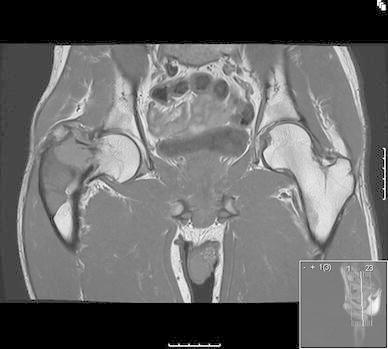
MRI scan of the pelvis demonstrating the fibrous dysplasia of the right proximal femur with the femoral neck fracture

Magnetic resonance imaging of the hip supported the diagnosis of FD (Fig. 2). The lesion extended from the proximal femoral diaphysis into the femoral neck. An intra-capsular femoral neck fracture had occurred through a cystic component of the lesion at the trans-cervical level.

The initial plan was to fix the fracture through closed reduction and internal fixation with cannulated screws. However, during the insertion of the third cannulated screw, the guide wire snapped and the proximal piece remained embedded in the hip joint. The patient was then referred to the senior author for further management. A decision was taken to remove all metalwork and correct the proximal femoral deformity whilst fixing the fracture.

### Operative treatment

**Fig. 3:**
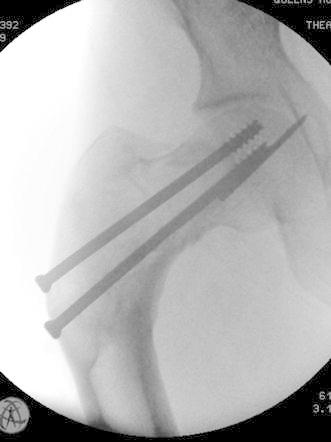
Antero-posterior radiograph of the right hip showing the broken guide wire during cannulated screw fixation of the fracture

The patient was placed supine on a traction table. A lateral skin incision was employed to remove the cannulated screws, and through the intra-capsular fracture site, the guide wire was removed by over-drilling around it, the fracture then reduced. A closing-wedge subtrochanteric femoral osteotomy was performed to correct the varus deformity. An eleven-hole AO Synthes proximal femoral plate (Synthes GmbH, Zuchwil, Switzerland) was then applied to achieve osteosynthesis at the osteotomy site, which incorporates screws to the femoral head. Allograft bone graft was used at the femoral neck at the site of the pathological fracture (Fig. 3).

**Fig. 4:**
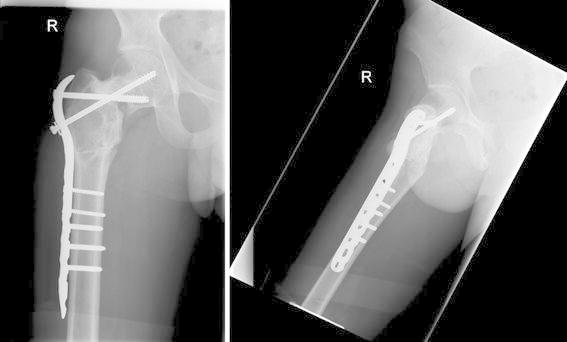
Antero-posterior and lateral radiographs of the right hip showing correction of the deformity and internal fixation of the femoral neck fracture, 12 months after the injury

The patient was rehabilitated non-weight bearing (NWB) for 6 weeks and then allowed to gradually progress to full-weight bearing (FWB). At 1-year follow-up, the patient was pain-free, walked with a normal gait with minimal weakness of the abductors. He demonstrated a full range of movement in the right hip with a reduced leg length discrepancy. Radiographs at 1 year showed union of the fracture and osteotomy site and a corrected femoral neck-shaft angle of 160°. A CT scan was not needed as the union was confirmed on the plain radiographs and the patient had no symptoms to indicate further imaging (Fig. 4).

## Discussion

Polyostotic fibrous dysplasia was first described by Lichtenstein [1], as a skeletal abnormality where the medullary cavities of multiple bones were filled with ‘gritty, greyish white fibrous tissue containing trabeculae of newly formed primitive bone’. It is now recognised that FD can occur as a solitary focus (monostotic form), in multiple bones (polyostotic form), or as part of the McCune–Albright syndrome. Mutations in the GNAS gene have been implicated in the pathogenesis of FD, but the precise molecular and cellular mechanism remains unknown [2].

Fibrous dysplasia lesions are commonly found in the proximal femur, where they can produce a progressive varus and bowing deformity from load transfer through weakened bone and repetitive microfracture. The shepherd’s crook deformity is associated with pain, limb shortening, limp and femoral neck fractures. Other common sites include the tibia, skull and ribs, although any bone can be affected. Radiographic features of FD include a cystic appearance characterised by areas of radiolucency, bone expansion endosteal scalloping and a ground-glass appearance with areas of homogenous or granular density [3]. Computerised tomography or MRI is useful to delineate the extent of the lesion and identify subtle fatigue fractures. A raised alkaline phosphatase and osteocalcin may be detected on blood analysis [4]. Histological examination demonstrates the replacement of normal cortical and cancellous bone and marrow with fibrous connective tissue containing spicules of disconnected woven bone. Malignant transformation into fibro- or osteo-sarcoma has been reported but is rare [5].

The management of FD remains a challenge. It is thought that the type of intervention should be guided by patient age, lesion characteristics (site, size and biological behaviour) and the presence of a significant deformity or fracture [6]. Asymptomatic lesions found incidentally in patients with the monostotic form are generally managed expectantly.

The use of second- and third-generation bisphosphonates has been advocated as a non-operative measure in selected patients. Clinical studies have demonstrated significant pain reduction, partial resolution of lesions and a lower incidence of stress fracture [7, 8]. However, long-term outcome data on large patient cohorts is not yet available.

Various surgical interventions have been described. Some procedures aim to provide symptomatic relief and limit the extent of the lesion, whilst others attempt to correct the varus deformity and provide mechanical support. Traditionally, patients with FD lesions in the proximal femur underwent intra-lesional curettage and filling with autogenous bone graft. Initial attempts with cancellous bone grafts resulted in early graft resorption and recurrence of the lesions [9, 10]. Better results have been achieved with cortical bone graft. Enneking and Gearen reported early graft resorption in only two cases from a series of 15 patients. In all other cases, good symptom relief was obtained, with no further fracture or progression of deformity. However, Guille et al. [11] in their long-term follow-up of 27 cases reported that all their bone grafts, cancellous and cortical, were resorbed and no lesion was eradicated or reduced. In addition, they argued that intra-lesional curettage disrupts the modest trabecular support that remains and further weakens the bone.

Individual case reports and case series have documented the treatment for shepherd’s crook deformities using corrective valgus osteotomies, combined with internal fixation using plates, screws or intramedullary nailing [12, 13]. Chen et al. [14] reported good pain relief and bone healing without recurrent deformity in two cases of polyostotic FD treated with corrective osteotomy and dynamic hip screws. Yang et al. [15] performed a valgus osteotomy, intra-lesional curettage, massive impaction allograft and intramedullary nailing in 14 hips. At a mean follow-up of 75 months, they reported good to excellent outcome in 12 patients, with no cases of recurrent fracture or progression of deformity. Although satisfactory results have been reported with intramedullary nailing, some surgeons feel that it is difficult to identify a good entry point in patients with a deformed and weakened proximal femur. Furthermore, studies have reported significant and progressive reductions in the neck-shaft angle in patients who underwent nailing without femoral neck stabilisation [16]. Critics of plate and screw fixation cite difficulties contouring the plate onto the deformed femur and the risk of stress-shielding and secondary deformity or fracture [17].

It has been argued that optimal correction of the varus deformity cannot be accomplished in a single procedure. A two-stage reconstruction has been described, with an initial subtrochanteric osteotomy and intramedullary nail fixation followed by an inter-trochanteric osteotomy and sliding nail plate 1 year later [18]. Sakurakichi et al. [19] reported a good outcome using the Ilizarov technique to correct shepherd’s crook deformity with pathological fractures in two patients. They argued that restoring accurate alignment is more important than resecting the lesion.

Patients with FD in the proximal femur may require total hip arthroplasty (THA) at a relatively young age. Sierra and Cabanel reported the outcome of 12 THA performed on 11 patients at a mean age of 44 years, with an average follow-up of 15.7 years [20]. They found that whilst THA offered satisfactory symptom relief, seven hips needed revision at a mean time of 12.5 years for loosening, with better surgical outcome in patients with monostotic disease in the proximal femur.

## Conclusion

This case highlights two important points: greater care is needed in decisions over fracture fixation as there is a higher risk of complications associated; when managing patients with acute femoral neck fractures and a shepherd’s crook deformity, it is advantageous to address the underlying deformity at the same time. A valgus osteotomy will correct the shepherd’s crook deformity, restore the mechanical alignment of the limb and reduce the risk of recurrent fracture. Furthermore, if the patient subsequently develops significant joint degeneration, a THA can be undertaken without need for further corrective surgery.
